# Relationship Between the Relative Age Effect and Lengths of Professional Careers in Male Japanese Baseball Players: a Retrospective Analysis

**DOI:** 10.1186/s40798-017-0090-3

**Published:** 2017-06-02

**Authors:** Hiroki Nakata

**Affiliations:** Department of Health Sciences, Faculty of Human Life and Environment, Nara Women’s University, Kitauoya-Nishi Machi, Nara city, 630-8506 Japan

**Keywords:** RAE, Baseball, Japan, Relative age effect

## Abstract

**Background:**

The mechanisms underlying the relative age effect in sport events have been investigated for more than two decades. The present study focused on the relationship between the relative age effect and lengths of professional careers among professional male Japanese baseball players.

**Methods:**

The birth dates of players and lengths of professional careers were collected from an official publication, and data were divided into four quarters (Q1: April–June; Q2: July–September; Q3: October–December; Q4: January–March of the following year) grouped by 3 years. Based on the data for Q4, the expected numbers for the lengths of professional careers were calculated for Q1, Q2, and Q3.

**Results:**

The number of players with professional careers of more than 19 years was significantly smaller in Q4 than in Q1, Q2, and Q3.

**Conclusions:**

The relative age effect among professional male Japanese baseball players was associated with the lengths of professional careers. Relative age appears to be a very important factor for the development of expertise among male Japanese baseball players and involves long-term disadvantages after becoming professional players.

## Key Points

• The present results indicate that a significant relative age effect exists not only in many junior sports but also in the lengths of professional careers after becoming a professional player.
• The number of Japanese male baseball players with professional careers of more than 19 years was markedly smaller in relatively young players than in relatively older players.
• Relative age involves long-term disadvantages after becoming professional players.

## Background

The relative age effect is regarded as a contributing factor to sporting success. For example, the Federation Internationale de Football Association (FIFA) uses a system for youth soccer with January 1 as the cut-off date to establish its age groups. Within the same age category, a difference of almost one full year may exist between the oldest and youngest participants. Therefore, relatively older children within a particular age group are more likely to achieve sporting success. This phenomenon has been called the relative age effect. Relatively older children have advantages in growth, biological maturity, and cognitive development [1]. In addition, relatively older children (athletes) have a greater opportunity to participate in competitions and, consequently, may enhance their psychological, technical, and tactical abilities, thereby supporting greater athletic development [2]. The relative age effect has been confirmed in many types of sports, including baseball [3, 4], soccer [5–8], tennis [9], cricket [10], basketball [4, 11], NASCAR [12], sumo wrestling [4], rugby [13], judo [2], ice hockey [14–17], and winter sports [18–21].

Moreover, several studies have examined the relative age effect from a historical perspective [8, 16, 22–25]. It generally takes several years or decades for a sport to gain popularity in a given country. Thus, historical analyses are needed in order to clarify the beginning of the relative age effect in a country and compare differences in the skew of this effect among generations.

The present study focused on how long the relative age effect continues into adulthood because most studies have focused on junior players, while, to the best of our knowledge, only a few studies have examined this topic. We previously reported that the relative age effect persisted among players older than 22 years of age when, theoretically, no physical advantage is expected for older players [26]. The relative age effect has been demonstrated in professional athletes who graduated university (college) at 22 years old; however, this relationship was weaker than that among those who graduated high school at 18 years old. Steingröver and colleagues [27] recently investigated whether relative age influenced career lengths in the National Basketball Association (NBA), National Hockey League (NHL), and National Football League (NFL). They showed that the number of matches played was significantly larger in relatively younger players than in relatively older players in the NHL. No significant differences were observed in career lengths in the NBA or NFL between relatively younger and older players.

The present study examined the relationship between the relative age effect and lengths of professional careers among professional male Japanese baseball players. Steingröver and colleagues [27] reported significant differences in career lengths between relatively younger and older players in the NHL; however, this relative age effect needs to be confirmed in other countries if universal factors are truly related to this effect. In other words, even if a significant relative age effect is observed in a country, the popularity and system of a sport differ among sports and countries. In Japan, a unique annual-age grouping has been applied since 1886, which is between April 1 and March 31 of the following year. Therefore, April 1 is the beginning of the “new year” (i.e., cut-off date), and this specific calendar follows an education system including elementary, junior high, and senior high schools and university (college), government, and companies. Sports calendars also follow this system. Thus, players born in April, May, and June are expected to have a relative age advantage. Grondin and Koren [23] reported that the relative age effect for baseball was more important in Japan than in the USA because large numbers of Japanese players were born during Q1 (April–June). Based on these backgrounds, a relative age effect was hypothesized to exist on the lengths of professional careers among Japanese professional male players.

## Methods

### Samples

The birth dates of players and lengths of professional careers were collected from an official publication [28]. Data from professional male Japanese baseball players (*N* = 4218 males) who played in Nippon Professional Baseball (NPB) and were born between 1911 and 1980 were analyzed. Baseball players were divided into four groups based on their month of birth: Q1 (April–June), Q2 (July–September), Q3 (October–December), and Q4 (January–March of the following year). Chi-squared tests were applied to each group according to the four quarters in order to assess the significance of deviations from the expected number of births in each quarter. The expected distribution was calculated based on national birth statistics for males during 1911–1980 in Japan (Ministry of Internal Affairs and Communications, the Statistics Bureau and the Director-General for Policy Planning of Japan). Professional male Japanese baseball leagues start in April and finish in October, with players being drafted in November. Foreign players were excluded because they had not passed through the Japanese school system.

Data for each quarter were then grouped by 3 years and categorized into seven groups (i.e., 1–3, 4–6, 7–9, 10–12, 13–15, 16–18, and 19 years). The reason for grouping by 3 years was that the sample size comprised more than 100 baseball players in each career year group. If data were grouped by 5 years (ex. 1–5, 6–10, 11–15, 16–20, and 21 years), the sample size for 21 years was less than 100 (Table 1). After grouping, based on the data of Q4, the expected numbers for the lengths of professional careers were calculated for Q1, Q2, and Q3. In this analysis, chi-squared tests were applied to Q1, Q2, and Q3 according to the lengths of professional careers in order to assess the significance of deviations from the expected number for the lengths of professional careers in Q4. Statistical tests were performed using computer software (SPSS for windows ver. 22.0). Significance was set at *p* ≤ 0.05.

**Table 1:** Number of players divided into lengths of professional careers based on quarters of the birth year

Year	Q1 (Apr–Jun)	Q2 (Jul–Sep)	Q3 (Oct–Dec)	Q4 (Jan–Mar)
1	221	202	134	129
2	145	143	99	109
3	128	115	86	89
4	90	89	61	42
5	84	75	57	60
6	82	88	35	39
7	72	60	36	43
8	85	70	44	50
9	61	50	39	23
10	54	42	28	48
11	51	61	38	32
12	41	31	29	32
13	49	35	28	29
14	41	29	34	28
15	36	33	17	23
16	28	19	22	11
17	15	22	7	13
18	21	18	13	19
19	14	7	11	4
20	7	9	7	4
21	8	4	1	1
22	4	6	4	1
23	4	3		2
24		1	1	
25			1	
26	1			
27				
28				
29	1			

## Results

Table 2 shows the birth date distribution of male baseball players and the general population. Chi-squared tests revealed significant relative age effects, indicating that the percentage of relatively older players in Q1 was clearly higher.

**Table 2:** Distribution of all players and the general population divided into quarters

	Q1 (Apr–Jun)		Q2 (Jul–Sep)		Q3 (Oct–Dec)		Q4 (Jan–Mar)		Total	*X* ^2^	*p*	*w*
	*n*	(%)	*n*	(%)	*n*	(%)	*n*	(%)	*n*			
Baseball players	1434	34.0	1212	28.7	832	19.7	831	19.7	4218	506.53	<0.001	0.347
[Expected]	928		1003		1008		1278					
General population	15,506,530	22.0	16,789,633	23.8	16,867,173	23.9	21,401,945	30.3	70,565,281			

The number in the second row shows the expected number of players obtained using the chi-squared test based on the number of the general population.

*n* number of players, *X* ^2^ chi^2^ value, *w* effect size

Table 3 shows the results of chi-squared tests for the lengths of professional careers in seven groups of 3 years. The distributions of Q1, Q2, and Q3 were significantly different from that of Q4 (Q1: *p* < 0.001, effect size 0.168; Q2: *p* < 0.001, effect size 0.156; Q3: *p* < 0.001, effect size 0.147). In addition, the distribution in six groups (i.e., from 1–3 to 16–18 years) was similar among Q1, Q2, Q3, and Q4. On the other hand, this difference was more prominent in those with a professional career spanning more than 19 years, showing that the number of baseball players with professional careers of more than 19 years was markedly smaller in Q4 than in Q1, Q2, and Q3 (Q1: *X* ^2^ = 19.8; Q2: *X* ^2^ = 8.9; Q3: *X* ^2^ = 14.0).

**Table 3:** Number of players grouped by 3 years for lengths of professional career

	1–3 years	4–6 years	7–9 years	10–12 years	13–15 years	16–18 years	19 years	Total	*p*	*w*
Q1 (Apr–Jun)	494	256	218	146	126	64	39	1343		
[Expected]	528	228	187	181	129	69	19	1343		
*X* ^2^	2.2	3.5	5.0	6.8	0.1	0.4	19.8	37.80	<0.001	0.168
Q2 (Jul–Sep)	460	252	180	134	97	59	30	1212		
[Expected]	477	206	169	163	117	63	18	1212		
*X* ^2^	0.6	10.4	0.7	5.3	3.3	0.2	8.9	29.48	<0.001	0.156
Q3 (Oct–Dec)	319	153	119	95	79	42	25	832		
[Expected]	327	141	116	112	80	43	12	832		
*X* ^2^	0.2	1.0	0.1	2.6	0.0	0.0	14.0	17.97	<0.001	0.147
Q4 (Jan–Mar)	327	141	116	112	80	43	12	831		

The number in the second row shows the expected number of players calculated from the number of players in Q4 (Jan–Mar)

*X* ^2^ chi^2^ value, *w* effect size

## Discussion

The present study investigated the relative age effect on the lengths of professional careers among male Japanese professional baseball players. The results revealed that the number of players with professional careers of more than 19 years was markedly smaller in Q4 than in Q1, Q2, and Q3. In addition, relative age was found to be a very important factor for the development of expertise among Japanese male baseball players and may involve long-term disadvantages after becoming professional players. In other words, even if relatively younger players became professional players, their talent may not be sufficient to continue for a long career such as more than 19 years, or they may be more likely to drop out of a professional career. This result was in contrast to previous findings showing the absence of a relative age effect in the NBA and NFL, and the favoring of relatively younger players in the NHL [27].

It is difficult to explain why relatively older baseball players have longer professional careers. One explanation is disadvantages in childhood. Relatively older players may have greater opportunities for selection and experience in childhood because they are naturally heavier, taller, stronger, and faster; have greater endurance; and are more coordinated than younger players during childhood [15], all of which translate into performance advantages in most sports [29]. This may lead to more long-term advantages for relatively older players in adulthood because of the development of self-confidence in childhood; however, these advantages are expected to become less apparent towards adulthood when physical maturity evens out. A second explanation is that this phenomenon is specific to Japan and Japanese professional male baseball players because many activities related to sports and academics are based on a unique cut-off date (April 1), which is not the case in other countries. Furthermore, as described in the “Background” section, the relative age effect for baseball is more important in Japan than in the USA [23]. These possibilities may interact. Further studies are needed in order to elucidate the mechanisms responsible for this phenomenon in more detail.

As a limitation of the present study, the lengths of professional careers may be associated with many factors other than the relative age effect. Baker and colleagues [30] reported that career lengths in Major League Baseball (MLB) were longer for infielders than for outfielders and catchers. Koz and colleagues [31] also showed a significant negative relationship between the draft round and games played in the NHL, NBA, and NFL and fielding players in MLB. However, the present study did not focus on the playing position or draft round. These factors need to be examined in more detail in future studies. In addition, in the present study, the significant relationship between the relative age effect and lengths of professional careers was only observed in Japanese male baseball players. Thus, this relationship needs to be examined in other sports including soccer, volleyball, Ekiden (a long-distance relay running race on roads), basketball, and sumo wrestling because these sports include a significant relative age effect among Japanese male athletes [4].

## Conclusions

The results of the present study provide additional information for elucidating the mechanisms underlying the relative age effect in professional sports. Our results suggest that the relative age effect in professional sports may be related to the lengths of professional careers.
